# Changes of lipid profiles after radical gastrectomy in patients with gastric cancer

**DOI:** 10.1186/s12944-015-0018-1

**Published:** 2015-03-21

**Authors:** Jin Won Lee, Eun Young Kim, Han Mo Yoo, Cho Hyun Park, Kyo Young Song

**Affiliations:** Department of Surgery, Seoul St. Mary’s Hospital, The Catholic University of Korea, College of Medicine, 222 Banpo-daero, Seocho-gu, Seoul 137-701 Korea

**Keywords:** Lipid profile, Gastrectomy, Gastric cancer, Weight loss

## Abstract

**Background:**

We investigated the changes of lipid profiles after radical gastrectomy.

**Methods:**

We analyzed the lipid-profile changes after radical gastrectomy in 144 patients with gastric cancer. Their lipid profiles, including total cholesterol (TC), triglyceride (TG), LDL-cholesterol (LDL), and HDL-cholesterol (HDL), were evaluated preoperatively as well as 6 and 12 months postoperatively. We compared the changes of lipid profile according to the reconstruction type and resection extent.

**Results:**

The TC level had decreased 6 months after surgery, and remained unchanged thereafter. The LDL level also had decreased 6 months after surgery, but had increased again after 12 months after surgery. The HDL level had increased 12 months after surgery, whereas the TG level was unchanged. In a comparison of the lipid levels according to the reconstruction type or resection extent, the HDL level significantly differed by reconstruction type 12 months after surgery: it was markedly higher in the total gastrectomy than in the subtotal gastrectomy group both 6 months and 12 months after surgery. Both the male gender and total gastrectomy were associated with probability of normalization of LDL after surgery.

**Conclusions:**

The lipid profiles including the TC, LDL and HDL levels were changed after radical gastrectomy; therefore, after this procedure, the lipid profiles of patients with hyperlipidemia should be evaluated.

## Background

Despite falling incidence rates, gastric cancer remains the most common malignancy in Korea and Japan [1]. Surgical resection is the mainstay for cure of patients with gastric cancer; the gastric volume reduction entailed in radical gastrectomy, however, leads to nutritional or metabolic disturbances [2] and impinges negatively on overall quality of life. The possible mechanisms of these problems have been known to include impaired food intake, malabsorption, impaired transit time, and decreased ghrelin levels. Gastric resection renders the mixing of food contents with digestive enzymes difficult, and curtails, thereby, lipid absorption. The consequently decreased food intake leads to weight loss, and human body expends proteins and lipid as supplemental sources of energy.

Changes in lipid profiles after bariatric surgeries for morbid obesity have been well established in clinical studies [3-8]. It has been shown that bariatric surgeries such as gastric banding, sleeve gastrectomy or Roux-en-Y gastric bypass provide effective remission of both morbid obesity and hyperlipidemia [2,3,5,9,10]. Lipid-profile changes are known to be associated with weight loss, malabsorption, decreased caloric intake, altered intestinal transit time, hormonal change, and diarrhea [11-14]. Such changes after gastrectomy for gastric cancer, however, have received relatively scant research attention. Lee et al. [2] found that cancer patients who had undergone gastric resection manifested lipid-profile, glucose-level and body-weight changes.

In the present study, therefore, we investigated the changes of lipid profiles after radical gastrectomy. The correlations of weight change and serum glucose level with lipid-profile change were also examined. According to our hypothesis that treatment type can determine changes of lipid profile, we evaluated the lipid profiles between different the different patient groups according to the type of reconstruction and the extent of resection.

## Materials and methods

### Patients and data collection

A total of 144 patients who had undergone curative radical gastrectomy at our hospital between 2011 and 2012 were enrolled. A complete evaluation including physical examinations, blood tests, chest and abdominal x-rays, upper-gastrointestinal endoscopy, endoscopic ultrasound, abdominal computed tomography and positron emission scanning was conducted both before and after surgery. Blood sampling following overnight fasting was performed to measure the total cholesterol (TC), triglyceride (TG), low-density lipoprotein (LDL), and high-density lipoprotein (HDL) lipid profiles preoperatively as well as 6 months and 12 months after surgery. Clinical data on age, gender, body mass index (BMI), stage, comorbidities, the operation types, and the extent of resection were reviewed.

The patients were divided into groups corresponding to both the extent of resection and the types of reconstruction, and the lipid-level changes among them were compared. Additionally, to elucidate the possible correlative clinicopathological factors leading to lipid-profile change, changes in body mass index (BMI) and fasting blood sugar (FBS) were analyzed preoperatively as well as 6 and 12 months after surgery. The resection extent was selected according to the Japanese gastric cancer treatment guidelines [15], which specify subtotal gastrectomy when a satisfactory proximal resection margin can be obtained, and total gastrectomy when tumors are located on the proximal side or along the greater curvature and harbor metastasis to the No.4sb lymph node. The reconstruction types, meanwhile, included Billroth I or II and Roux-en-Y. Finally the relationships between the metabolic parameters and the perioperative clinical factors were analyzed. Approval for this study was obtained from our Institutional Review Broad (IRB KC14RISI0482).

### Post-gastrectomy diet schedule

We have critical pathway including diet schedule after radical gastrectomy for patients with gastric cancer. The patients were taught to keep in mind that they should have half amount of soft meal six times a day for 1 month after surgery. And thereafter they can eat regular meals but we usually recommend to eat small, frequent meals(about two hourly) for quite a long time.

### Statistical analysis

Continuous data were expressed as the mean ± standard deviation. For inter-subgroup comparison, the mean values were analyzed using repeated measures ANOVA (adjusted for preoperative values) with a post-hoc Holm-Bonferroni method test. If Mauchly’s test of sphericity was violated, the Greenhouse-Geisser correction was applied. All of the parameters were subjected to logarithmic transformation, which transformed values which were then converted to their original units by back-transformation based on exponentiation. The independent factors associated with correction of lipid profiles were evaluated by statistics carried out using univariate and multivariable logistic regression models. A final data analysis was performed using SPSS software (version 12.0; SPSS, Chicago, Ill). The critical *p* value for significance was set at 0.05.

## Results

**Figure 1:**
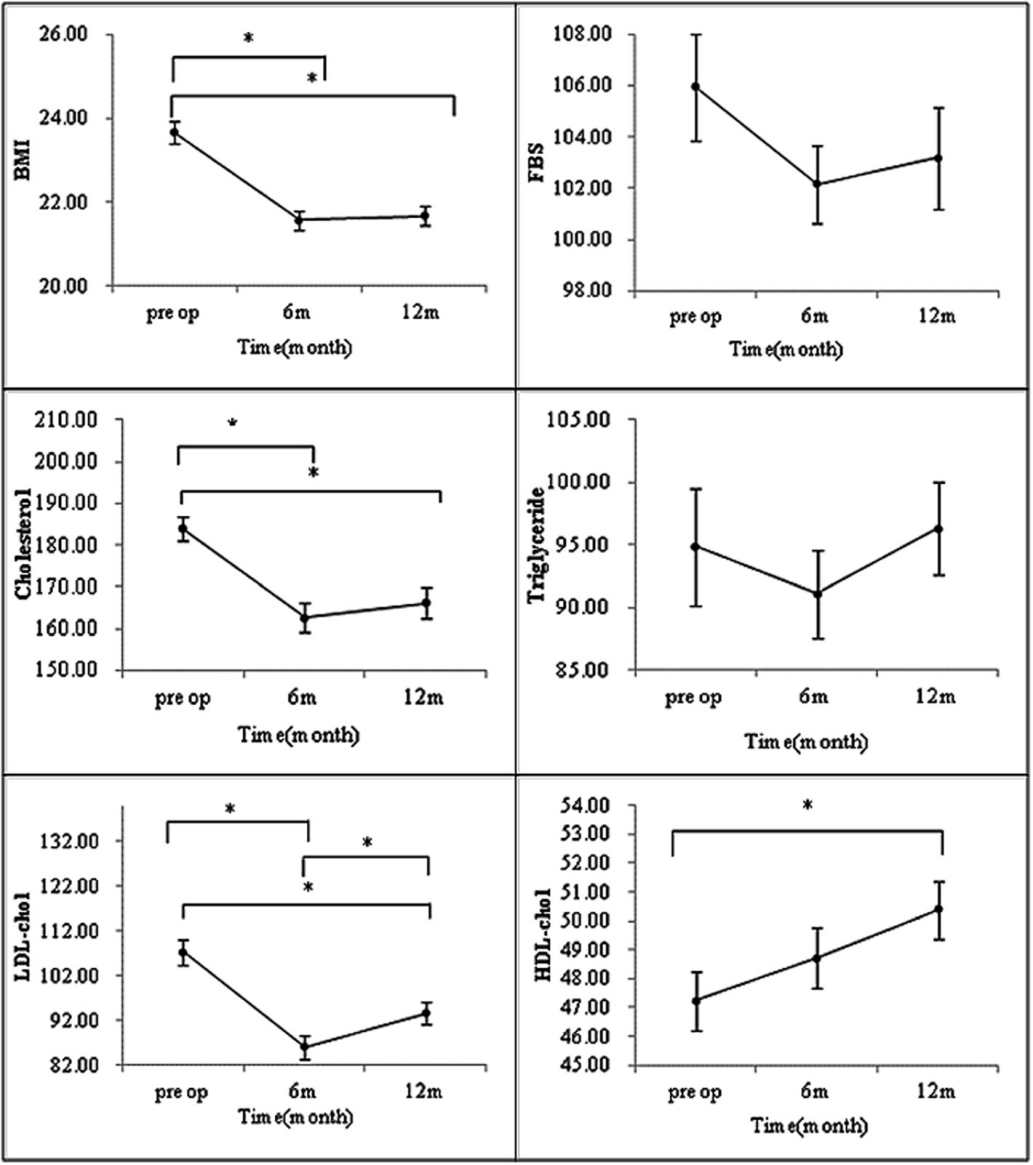
Alterations of metabolic parameters 6 and 12 months after gastrectomy in all cohorts (*P < 0.05).

The baseline characteristics of the patients are listed in Table 1. Among the 144 patients, 92 (64%) were men, whose mean BMI mean levels of TC, TG, HDL and LDL were 23.67 kg/m^2^, 183.89 mg/dl, 94.87 mg/dl, 47.23 mg/dl and 107.25 mg/dl, respectively. Over the course of the 12-month follow, the BMI and lipid profile changed at particular time points (Table 2) (Figure 1). The mean TC level had significantly decreased 6 months postoperatively, from 183. 89 ± 2.84 to 162.66 ± 3.56 mg/dl (P < 0.0001), but remained stagnant thereafter. The mean HDL level had increased significantly 12 months postoperatively, from 47.23 ± 1.01 to 50.40 ± 1.00 mg/dl(P < 0.0012). There was also a significant decrease in the LDL level 6 months postoperatively, from 107.25 ± 2.75 to 86.00 ± 2.59 mg/dl (P < 0.0001), followed by a significant increase 12 months postoperatively to 93.65 ± 2.43 mg/dl(P < 0.0001). However, no significant changes of TG level were observed either 6 or 12 months after surgery.

**Table 1:** **Baseline characteristics of patients**

**Variables**	**No. (%) (n = 144)**
Sex	
Male	92(63.89)
Female	52(36.11)
Age (years)	
mean ± SD	58.63 ± 9.86
median(range)	60(36–81)
Stage	
I	110(76.39)
II	18(12.5)
III	16(11.11)
Comorbidity	
No	51(35.42)
Yes	93(64.58)
Diabetes	
No	116(80.56)
Yes	28(19.44)
Hypertension	
No	103(71.53)
Yes	41(28.47)
Smoking	
No	57(39.58)
Yes	87(60.42)
Operation approach	
Open	114(79.17)
Laparoscopic	30(20.83)
Resection extent	
Total	38(26.39)
Subtotal	106(73.61)
Reconstruction	
B-I	21(14.58)
B-II	79(54.86)
RY	44(30.56)

B-I, Billorth I; B-II, Billroth II; RY, Roux en Y.

**Table 2:** **The changes in metabolic parameters at the end of the 12 month study**

					**post hoc Holm–Bonferroni method test**		
	**pre op**	**6 month**	**12 month**	***p*** **value**	**(pre-6 m)** ***p*** **value**	**(pre-12 m)** ***p*** **value**	**(6 m-12 m)** ***p*** **value**
TC	183.89 ± 2.84	162.66 ± 3.56	166.22 ± 3.76	<0.0001	<0.0001	<0.0001	0.4042
TG	94.87 ± 4.66	91.14 ± 3.51	96.29 ± 3.70	0.3790			
HDL	47.23 ± 1.01	48.73 ± 1.05	50.40 ± 1.00	0.0225	0.1235	0.0012	0.0866
LDL	107.25 ± 2.75	86.00 ± 2.59	93.65 ± 2.43	<0.0001	<0.0001	<0.0001	<0.0001
weight	62.61 ± 0.87	57.08 ± 0.78	57.30 ± 0.79	<0.0001	<0.0001	<0.0001	0.3465
BMI	23.67 ± 0.25	21.58 ± 0.23	21.67 ± 0.23	<0.0001	<0.0001	<0.0001	0.3483
FBS	105.94 ± 2.09	102.17 ± 1.53	103.20 ± 1.98	0.2435			

Data are presented as mean ± SD, TC, total cholesterol; TG, triglyceride; HDL, high-density lipoprotein cholesterol; LDL, low-density lipoprotein cholesterol; BMI, body mass index; FBS, fasting blood sugar.

**Figure 2:**
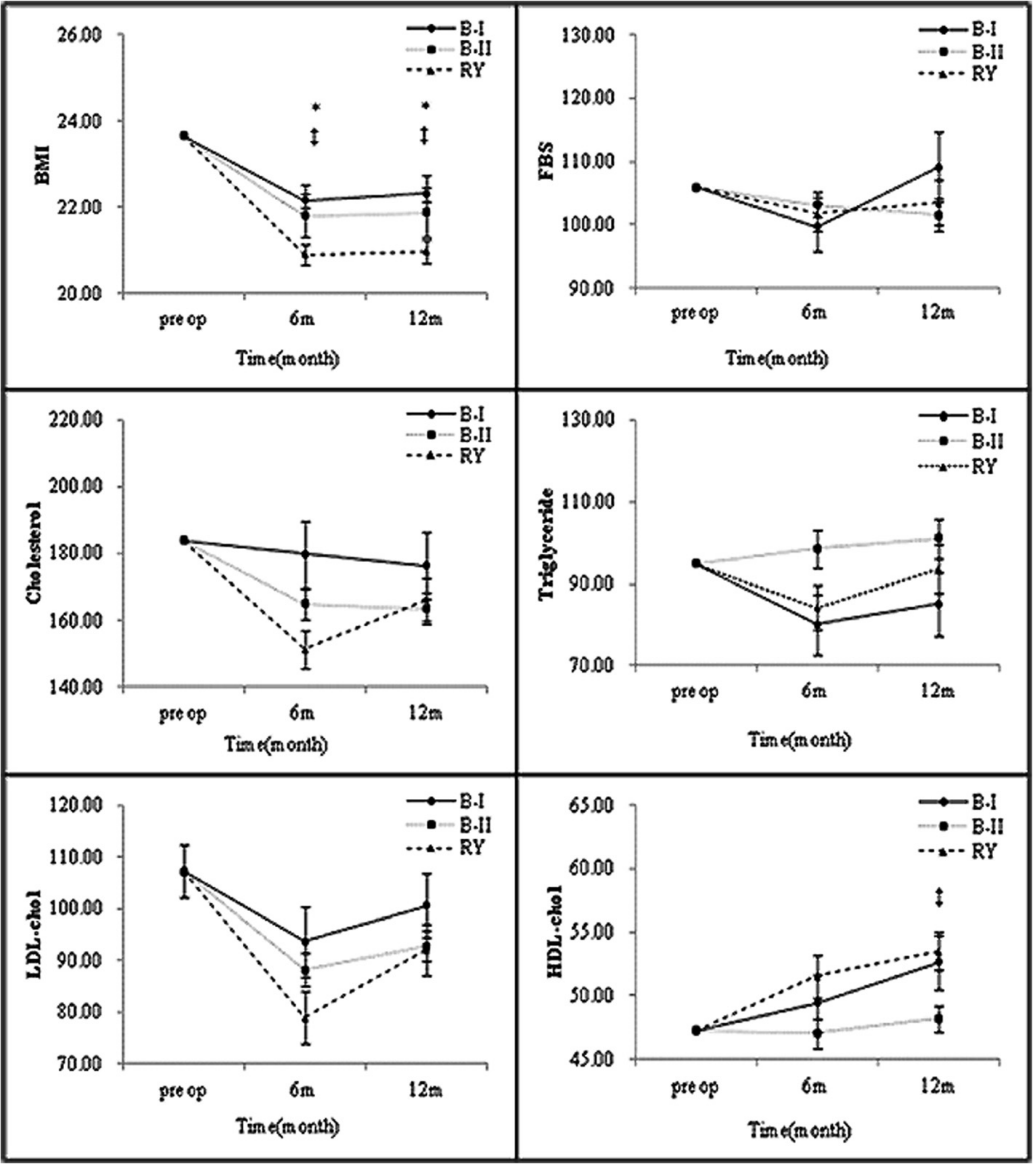
Subgroup analysis of metabolic parameters 6 and 12 months after gastrectomy by reconstruction type (*B-I vs. RY, †B-I vs. B-II, and ‡B-II vs. RY (P < 0.05)).

We next compared the lipid-profile changes according to the reconstruction type. No differences in TC, TG or LDL level were apparent for the reconstruction types either 6 or 12 months after surgery. The level of HDL, by contrast, was significantly lower in patients who had undergone Roux-en-Y (RY) reconstruction than in those who had undergone Billroth I or II 12 months after surgery (P = 0.0098) (Figure 2).

**Figure 3:**
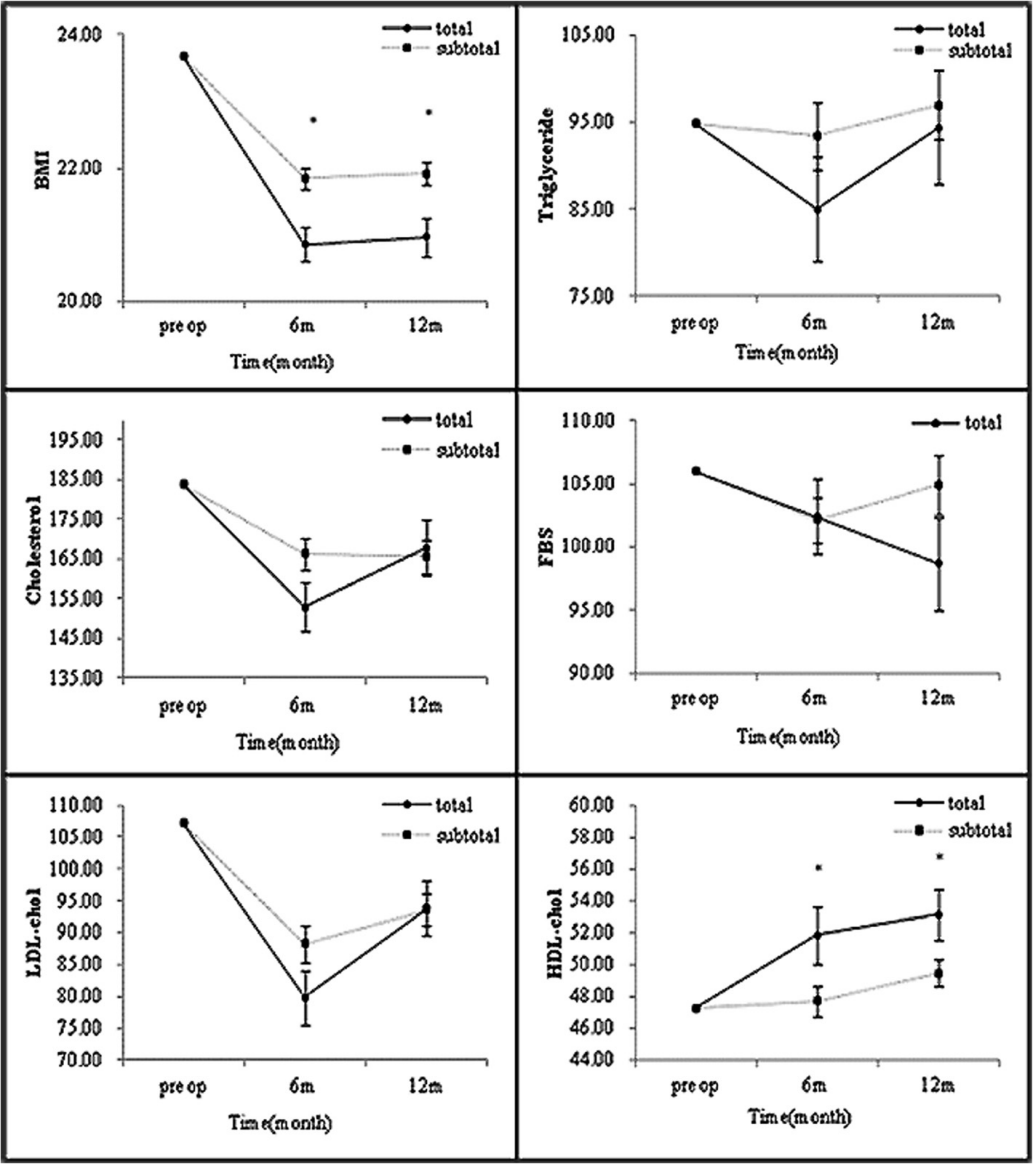
Subgroup analysis of metabolic parameters 6 and 12 months after gastrectomy by resection extent (*P < 0.05).

We also compared the lipid-profile changes according to the resection extent. Once again, no differences in TC, TG or LDL level were evident between the total and subtotal gastrectomy cases either 6 or 12 months after surgery. The level of HDL, however, was significantly lower in patients who had undergone total gastrectomy than in those who had undergone subtotal gastrectomy both 6 (P = 0.0422) and 12 months (P = 0.0464) after surgery (Figure 3).

We subsequently analyzed the independent factors associated with correction of abnormal lipid profile using logistic regression models. There were 49, 55, 120 and 93 patients who showed abnormal preoperative TC, TG, HDL and LDL levels, respectively (Table 3). There were no independent clinical factors associated with the correction of abnormal TC, TG or HDL. But for the correction of abnormal LDL, there were significant associations with gender [OR = 0.39, 95% CI = 0.153-0.847, P = 0.0369], extent of resection [OR = 0.36, 95% CI = 0.14-0.97, P = 0.0438] and type of reconstruction [OR = 0.4.47, 95% CI = 1.01-19.68, P = 0.0143] on multivariate analysis (Table 4).

**Table 3:** **Changes of lipid profile between preoperative period and 6 month postoperatively**

	**Normal → Normal**	**Normal → Abnormal**	**Abnormal → Abnormal**	Abnormal → Normal*
TC	83	12	12	37
TG	72	17	22	33
HDL	11	13	104	16
LDL	44	7	45	48

Reference levels; total cholesterol (TC) <200 mg/dl, triglycerides (TG) 40–120 mg/dl, HDL-cholesterol (HDL) >60 mg/dl, LDL-cholesterol (LDL) <100 mg/dl.

*The number of patients whose lipid profile were normalized at 6 months after surgery.

**Table 4:** **Analysis of clinicopathologic factors associated with resolution of LDL-cholesterol at 6 month**

	**Abnormal → Abnormal (n = 45)**	**Abnormal → Normal (n = 48)**	**OR (95% CI)**	***p*** **value**	**Adjust OR (95% CI)**	***p*** **value**
Sex						
Male	21(46.67)	34(70.83)	1		1	
Female	24(53.33)	14(29.17)	0.36(0.153–0.847)	0.0192	0.39(0.16–0.95)	0.0369
Age (years)						
Mean(SD)	58.71(8.56)	57.85(11.28)	1			
Median(range)	61.00(38.00–73.00)	58.00(36.00–81.00)	0.99(0.95–1.03)	0.6786		
Stage						
IA	37(82.22)	23(47.92)	1			
IB	2(4.44)	5(10.42)	4.02(0.72–22.47)	0.9480		
IIA	1(2.22)	3(6.25)	4.83(0.47–49.22)	0.9534		
IIB	2(4.44)	6(12.5)	4.83(0.90–25.97)	0.9534		
IIIA	1(2.22)	3(6.25)	4.83(0.47–49.22)	0.9534		
IIIB		5(10.42)	Inf	0.9148		
IIIC		2(4.17)	Inf	0.9435		
Comorbidity						
No	23(51.11)	19(39.58)	1			
Yes	22(48.89)	29(60.42)	1.60(0.70–3.63)	0.2654		
DM						
No	42(93.33)	39(81.25)	1			
Yes	3(6.67)	9(18.75)	3.23(0.82–12.81)	0.0952		
HBP						
No	35(77.78)	36(75)	1			
Yes	10(22.22)	12(25)	1.17(0.45–3.05)	0.7529		
Op approach						
Open	35(77.78)	40(83.33)	1			
Laparoscopic	10(22.22)	8(16.67)	0.70(0.25–1.97)	0.4992		
Resection extent						
Total	8(17.78)	19(39.58)	1		1	
Subtotal	37(82.22)	29(60.42)	0.33(0.13–0.86)	0.0234	0.36(0.14–0.97)	0.0438
Reconstruction						
B-I	8(17.78)	4(8.33)	1		1	
B-II	29(64.44)	22(45.83)	1.52(0.41–5.69)	0.3491	1.38(0.36–5.32)	0.3663
RY	8(17.78)	22(45.83)	5.50 (1.29–23.39)	0.0050	4.47(1.01–19.68)	0.0143

Statistics were carried out using univariate and multivariable logistic regression. Use of univariate analyses to select variables for multivariable models (P <0.05). B-I, Billorth I; B-II, Billroth II; RY, Roux en Y.

**Figure 4:**
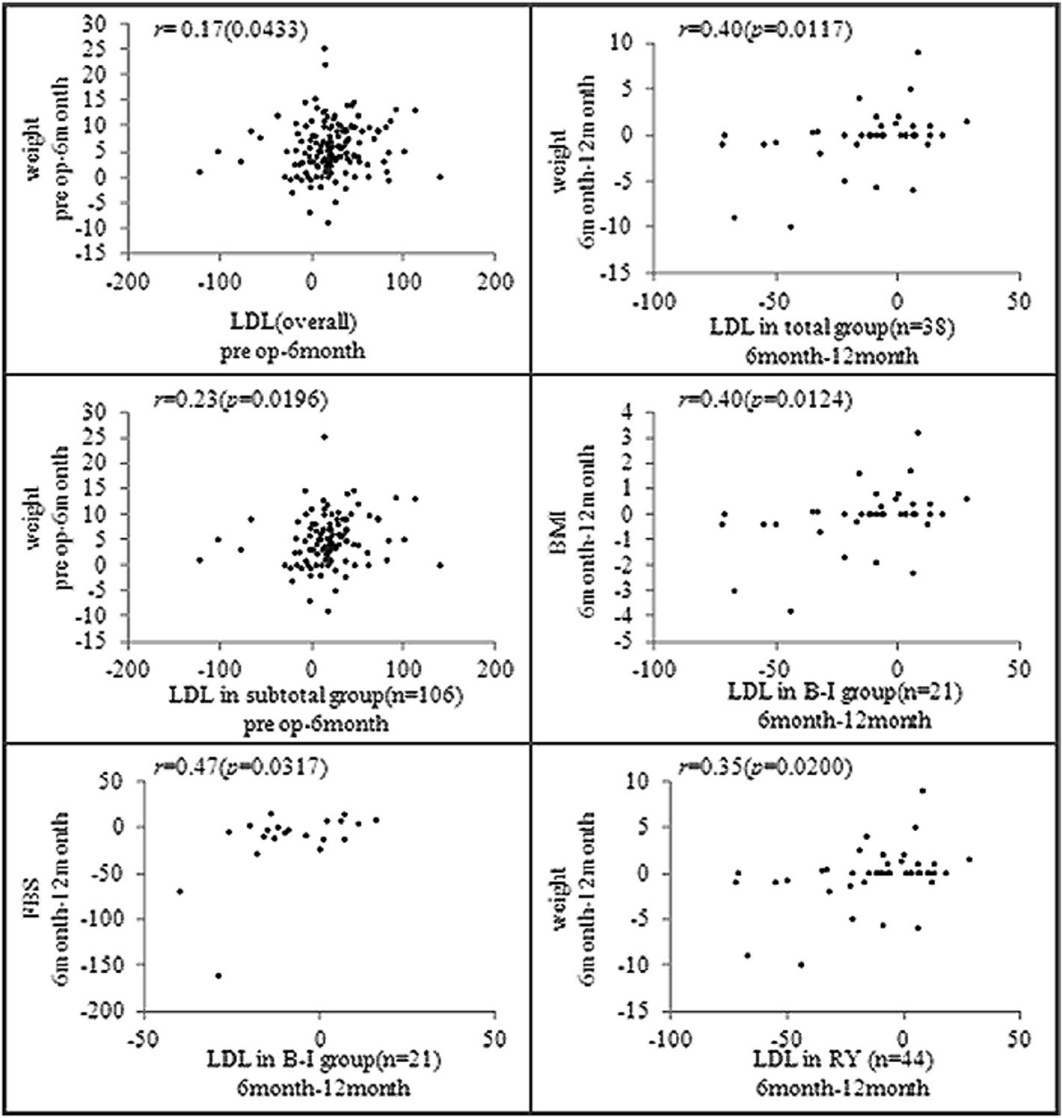
**Correlations between baseline characteristics and LDL-cholesterol.** The data are presented as Spearman’s rank correlation coefficients (*p* values).

Finally, we examined the correlations between the patients’ LDL levels and their baseline characteristics by Spearman correlation analysis. Changes of LDL were associated with weight loss in all patients 6 months after surgery (r = 0.17, P = 0.0433), and this association was significant in the patients who had undergone subtotal gastrectomy (r = 0.23, P = 0.0196). In the patients who had undergone total gastrectomy, the change of LDL from 6 to 12 months postoperatively also was related to weight loss (r = 0.40, P = 0.0117). The change of LDL from 6 to 12 months postoperatively in the patients who had undergone B-I reconstruction, meanwhile, was associated with both BMI (r = 0.40, P = 0.0124) and FBS (r = 0.47, P = 0.0317). The LDL change from 6 to 12 months postoperatively in those who had undergone RY reconstruction was associated with weight loss(r = 0.35, P = 0.02) (Figure 4).

## Discussion

A number of patients who have undergone gastrectomy for gastric cancer have experienced metabolic changes. These include weight loss, fat malabsorption, hormonal change and disturbance of carbohydrate metabolism. The pathogenesis of these changes is complex, involving potentially interacting factors such as type of operation, extent of gastric resection, and patients’ eating habits [16,17]. In the present study, we evaluated the lipid-profile trends in gastric cancer patients who had undergone gastrectomy and hypothesized that lipid-profile changes would be associated with several factors such as reconstruction type, resection extent, and others.

With respect to the extent of gastric resection, statistically significant differences in the changes of BMI and HDL cholesterol levels were noted between the two groups. Although the BMI in both groups showed a downward trend, the change was more marked in the patients who had undergone total gastrectomy. Correspondingly, the HDL cholesterol increased in both groups with time, but the total gastrectomy group showed a more significant change than did the subtotal group. After total gastrectomy, the loss of gastric reservoir space reduces caloric intake, whereas after subtotal gastrectomy, reservoir function can be restored, with food intake gradually becoming normalized. Still, Liedman et al. [18] reported that post-gastrectomy weight loss is due to loss of fat, and concluded that malabsorption of fat does not differ between those having a gastric remnant and those who do not. Jagat et al. [19] found that weight loss after subtotal gastrectomy does not significantly differ according to resected stomach volume, which was greater in their patients who had a higher preoperative BMI.

Taking the types of reconstruction into account, BMI decreased and HDL cholesterol increased in all three groups, though the change in the RY group was more significant than those in the B-I and B-II groups. Tanaka et al. [20] reported that visceral fat loss after RY is larger than that after B-I, due to an association of duodenal bypass with loss of visceral fat. Wang et al. [10] explained that B-I reconstruction, which carries anatomic and physiologic advantages, is associated with postoperative weight recovery. Hyroyuki et al. [21] emphasized the superiority of B-I reconstruction to RY in terms of fat digestive and absorption functions, noting that B-I reconstruction allows for the physiologic passage of ingested food through the duodenum.

In our analysis of the association between clinicopathologic factors and lipid-profile resolution, the male gender, total gastrectomy and RY reconstruction were found to be correlated with the probability of LDL resolution after surgery (Table 4). Also, a Spearman’s correlation analysis showed that the LDL changes were associated with weight reduction (Figure 4). Nguyen et al. [17] reported that weight reduction after bariatric surgery is very effective for achieving TC, TG, LDL declines with increased favorable lipoprotein (HDL).

There have been some suggestions that hyperlipidemia can result in depression of cellular immunity and an increased possibility of malignant transformation [22]. Dilman et al. [23] reported that disturbance of lipid metabolism causes tumor growth and hinders DNA repair, and suggested dietary and pharmacological means of lipid metabolism correction in cancer prophylaxis and therapy. Kim et al. [24] reported that because hypercholesterolemia is a risk factor for gastric dysplasia as well as an important prognostic factor, control of lipid profiles can improve the therapeutic efficacy of gastric cancer treatments. In this light, changes of lipid profiles after radical gastrectomy for gastric cancer should be closely monitored, and patients with persistent hyperlipidemia should be considered for further management such as dietary modification or administration of lipid-lowering agents. In any case, further study on the relationship between post-gastrectomy metabolic change and oncologic results such as recurrence and survival is required.

Recently, early diagnosis and optimal treatment chance for gastric cancer have been increased. As a result, specific concerns were raised in quality of life after radical gastrectomy for gastric cancer as the number of long-term survivors has increased. Furthermore, the incidence of obesity and hyperlipidemia has been steadily increasing in Asian countries due to the westernized dietary habit and decreased physical activity. However there have been few observations about change of lipid profile in patient with gastric cancer who underwent surgery. It has been well known that obesity and hyperlipidemia is associated with poor health-related quality of life. So we think that regular follow-up of lipid profile and weight may provide important information about metabolic status of the patients and might guide long-term follow-up strategies.

This study has several limitations. First, there might have been a selection bias rooted in the fact that this was a retrospective analysis. Second, the enrolled patients were not metabolically even or uniform; and moreover, they had gastric cancers of differing histology, stage, and location, which oncologic variety might have had an effect on the results. Third, the effect of gut hormones was not supported by the laboratory examination results. Finally, it would have been helpful if we had checked apolipoprotein A1 and B. Apolipoprotein A1 and B are the major protein components of HDL and LDL, respectively, and are well known biomarkers for prediction of cardiovascular diseases. We should’ve checked these factors, however, we do not routinely measure them for patients with gastric cancer. A large-scale prospective study including these laboratory test is needed to evaluate the mechanism of these results.

## Conclusion

In conclusion, 12 months after surgery, significant weight loss and changes in the levels of TC, LDL and HDL were noted, whereas the level of TG remained unchanged. The HDL level differed in accordance with both the reconstruction type and the resection extent. Total gastrectomy and RY reconstruction had the more significant impacts on the lipid-profile changes. The male gender, total gastrectomy and RY reconstruction were all associated with the probability of correction of abnormal LDL after surgery, and the change of LDL level was correlated with weight loss. Close follow up and strict control of lipid levels are recommended for gastric cancer patients with hyperlipidemia after radical gastrectomy.
